# Supplementary material for: Elevated moisture stimulates carbon loss from mineral soils by releasing protected organic matter
Source: Nat Commun. 2017 Nov 24;8:1774. doi: 10.1038/s41467-017-01998-z (PMC5701196; doi:10.1038/s41467-017-01998-z)
Supplement: Supplementary file 1 — Supplementary Information [file 41467_2017_1998_MOESM1_ESM.pdf]

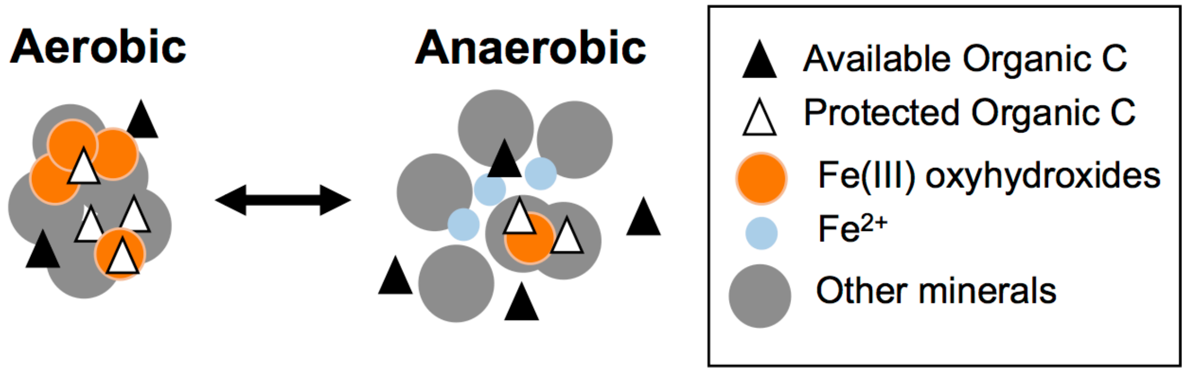

Supplementary Figure 1. Schematic illustration of the influence of reducing conditions on organic C bound to Fe. Under aerobic conditions, a significant fraction of soil C may be associated with Fe(III) oxyhydroxides. Under anaerobic conditions, a portion of those Fe(III) phases may be reduced by microbes to Fe^{2+}, releasing organic C in colloidal and soluble forms which are readily available or can be rapidly depolymerized for microbial assimilation.

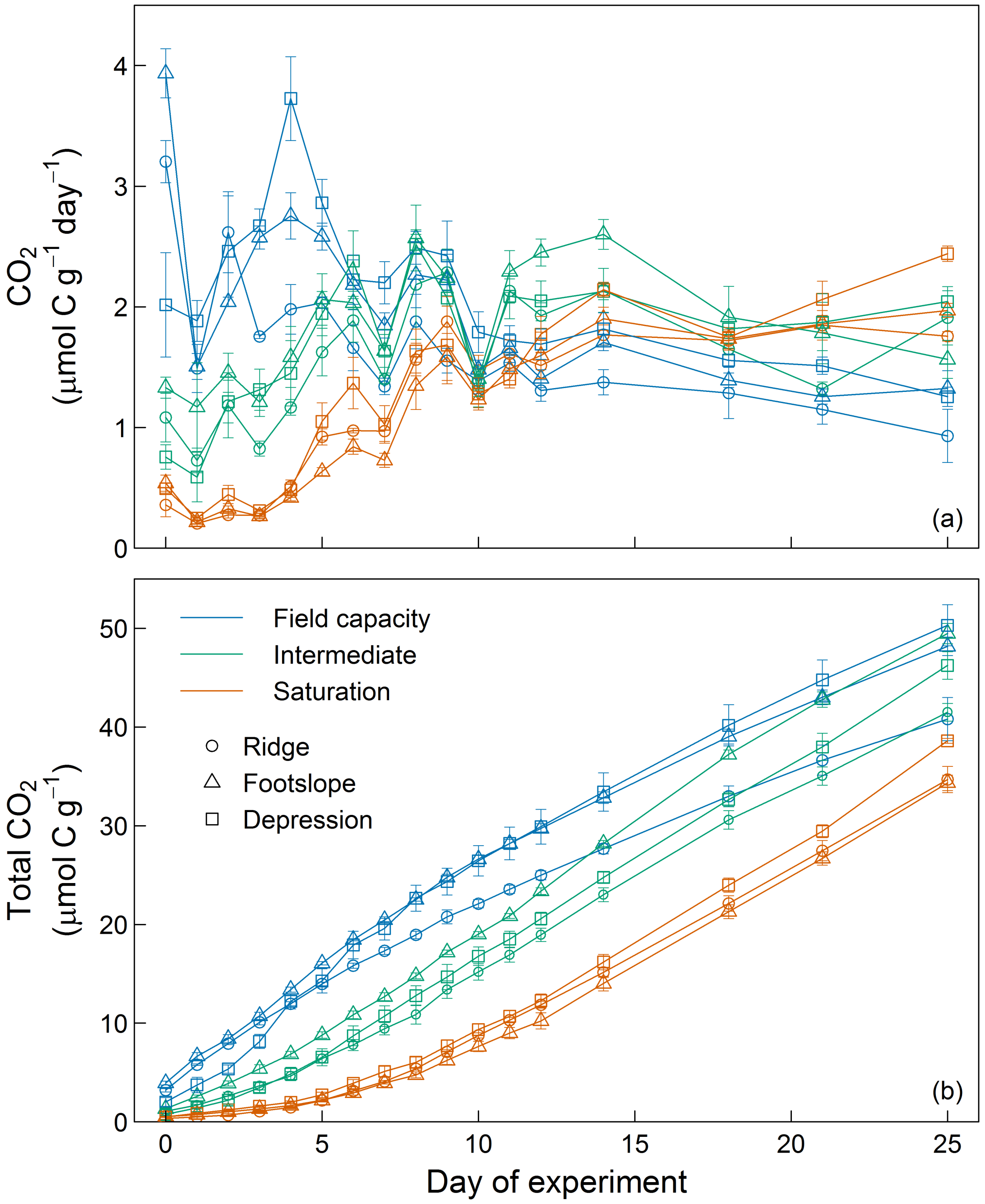

Supplementary Figure 2. Initial CO_2 production from three Mollisols incubated under three soil moisture levels. (a) CO_2 production rate; (b) cumulative CO_2 production. The error bars indicate s.e.m. ($n = 4$).

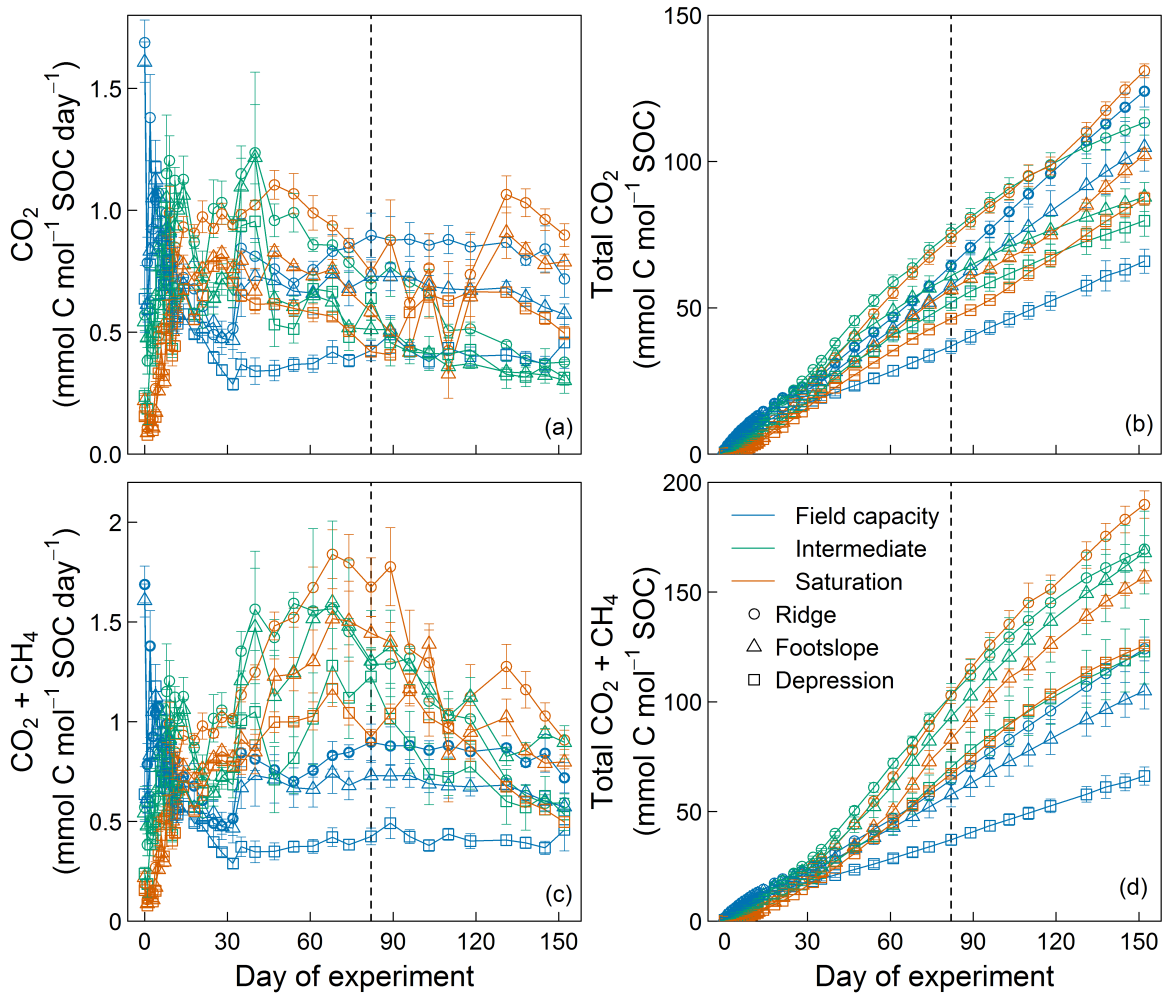

Supplementary Figure 3. Carbon mineralization normalized by soil C from three Mollisols incubated under moisture levels at and above field capacity. (a) CO_2 production rate; (b) cumulative CO_2 production; (c) total C mineralization rate (CO_2 + CH_4); (d) cumulative total C mineralization (CO_2 + CH_4). The vertical dashed line indicates when gradual drainage was initiated in the saturated soils. The error bars indicate s.e.m. (n = 4).

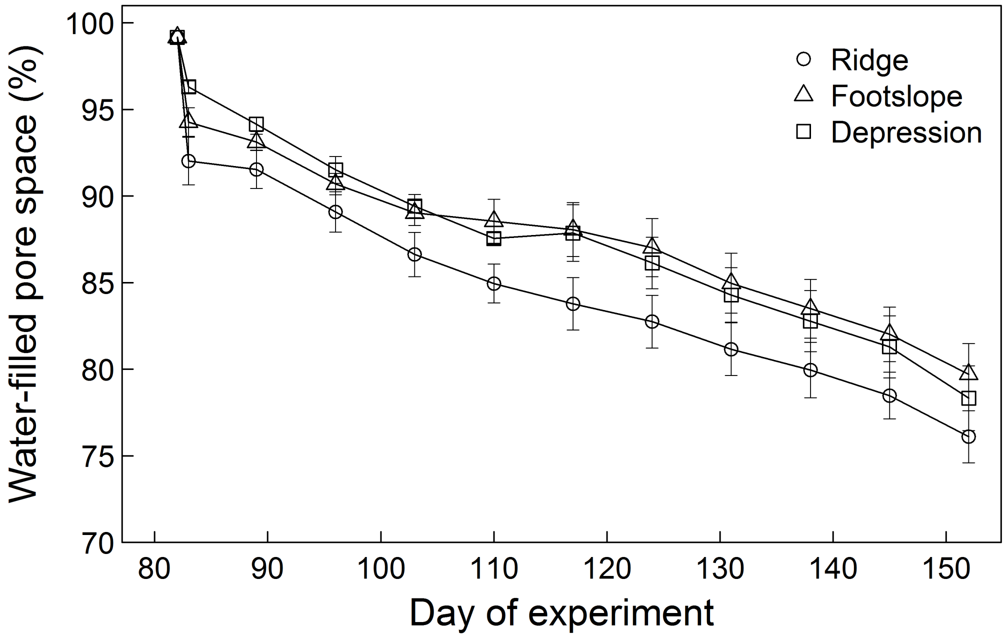

Supplementary Figure 4. Changes in soil moisture in three Mollisols incubated under saturated treatment during drainage. The error bars indicate s.e.m. ($n = 4$).

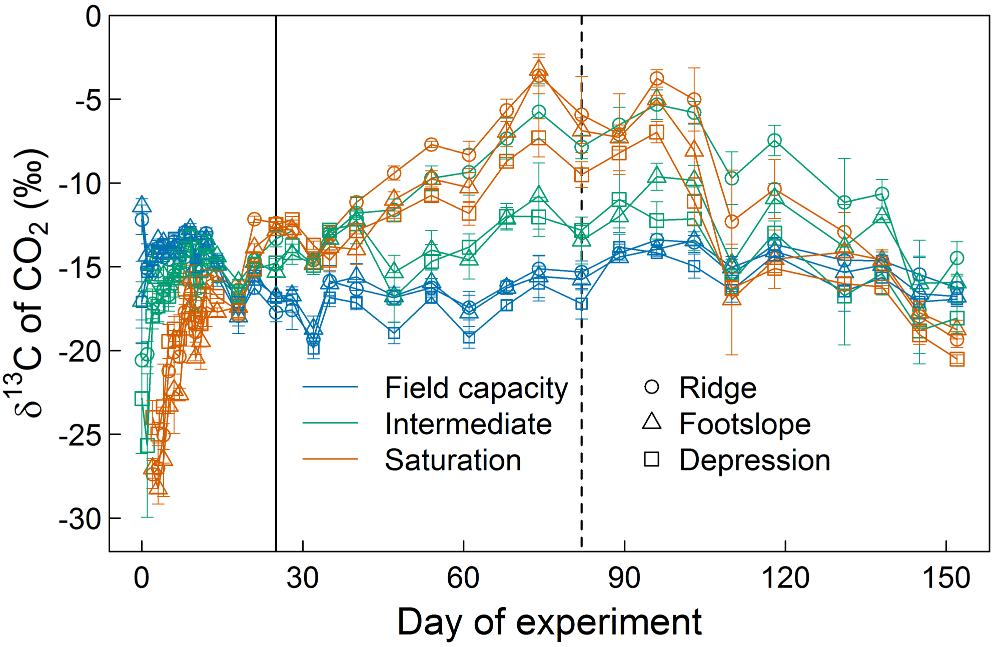

Supplementary Figure 5. Carbon isotope ratios of CO_2 produced from three Mollisols incubated under moisture levels at and above field capacity. The vertical solid line indicates when CH_4 production began in the intermediate and saturated treatments, leading to increased $\delta^{13}C$ values of CO_2 due to fractionation associated with methanogenic CO_2 reduction, and enrichment of $\delta^{13}C$ in the residual CO_2. The vertical dashed line indicates when gradual drainage was initiated in the saturated soils. The error bars indicate s.e.m. (n = 4).

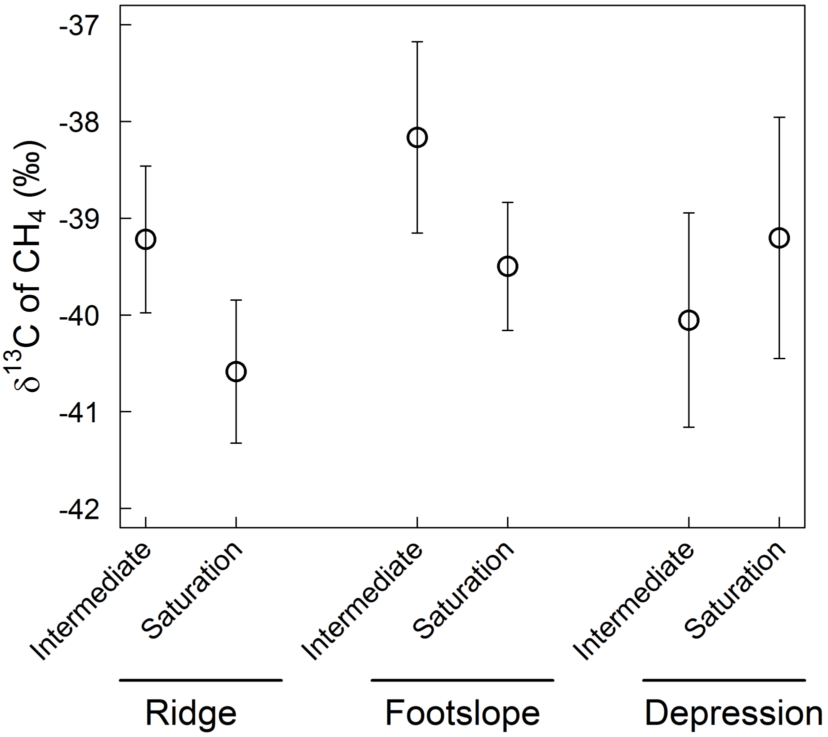

Supplementary Figure 6. Carbon isotope ratios of CH_4 produced from three Mollisols incubated under moisture levels at and above field capacity. Circles are means of four sampling dates, and the error bars indicate s.e.m. (n = 4).

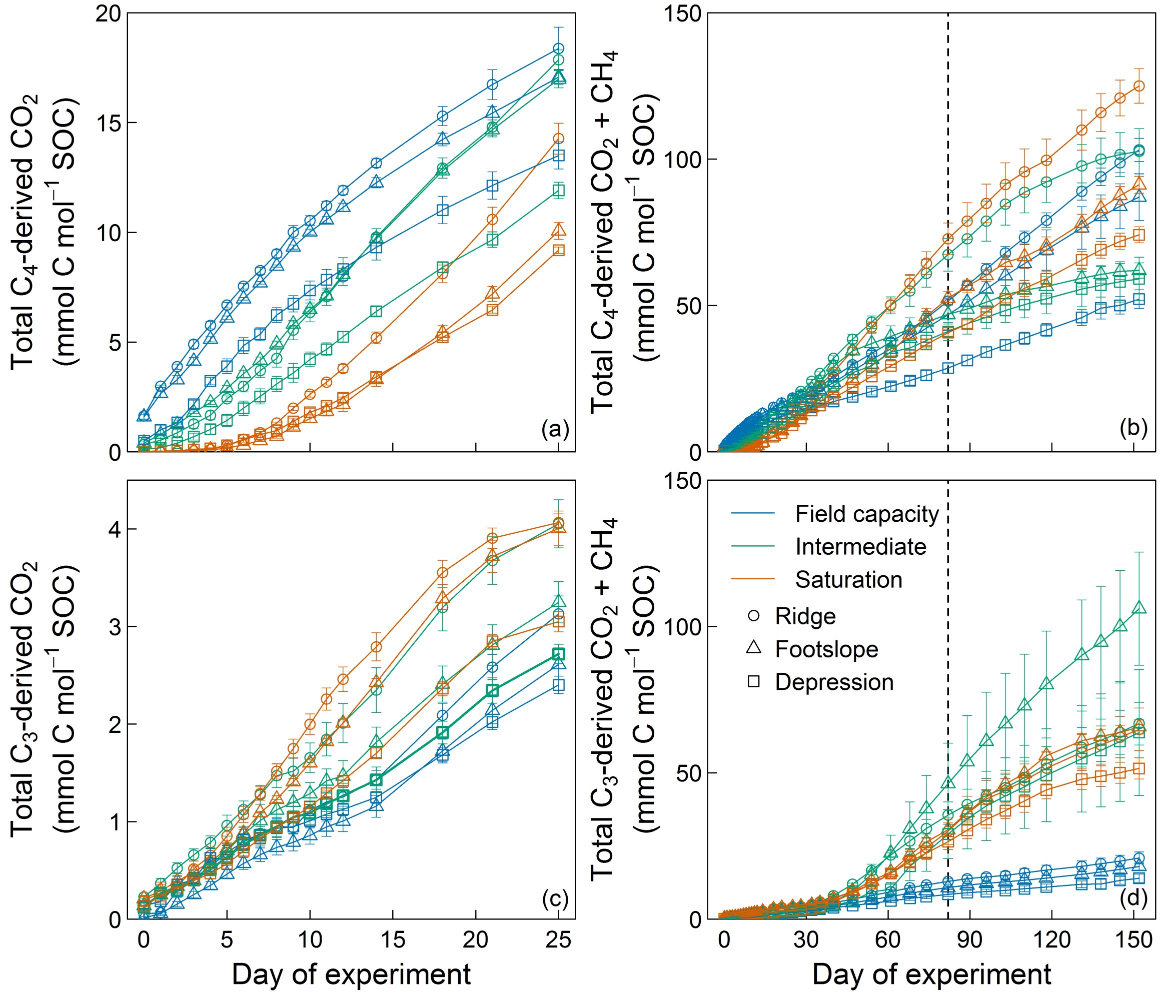

Supplementary Figure 7. Cumulative mineralization of different C sources normalized by soil C in three Mollisols incubated under moisture levels at and above field capacity. (a, c) Short-term cumulative mineralization of C_4-derived C (a) and C_3-derived C (c) respired as CO_2; (b, d) long-term cumulative mineralization of C_4-derived C (b) and C_3-derived C (d) respired as CO_2 and CH_4. The vertical dashed line indicates when gradual drainage was initiated in the saturated soils. The error bars indicate s.e.m. (n = 4).

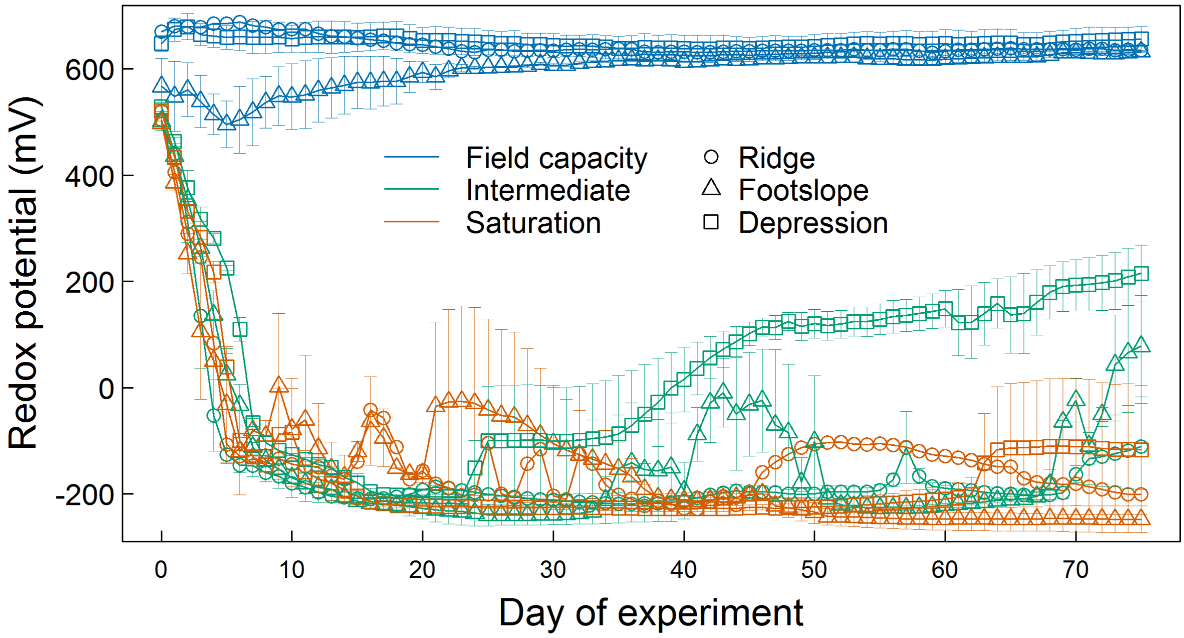

Supplementary Figure 8. Temporal variations of redox potential in three Mollisols incubated under three soil moisture levels. Data are expressed relative to the standard hydrogen electrode. Symbols represent daily means. The error bars indicate s.e.m. (*n* = 2 except for ridge soil (*n* = 1)).

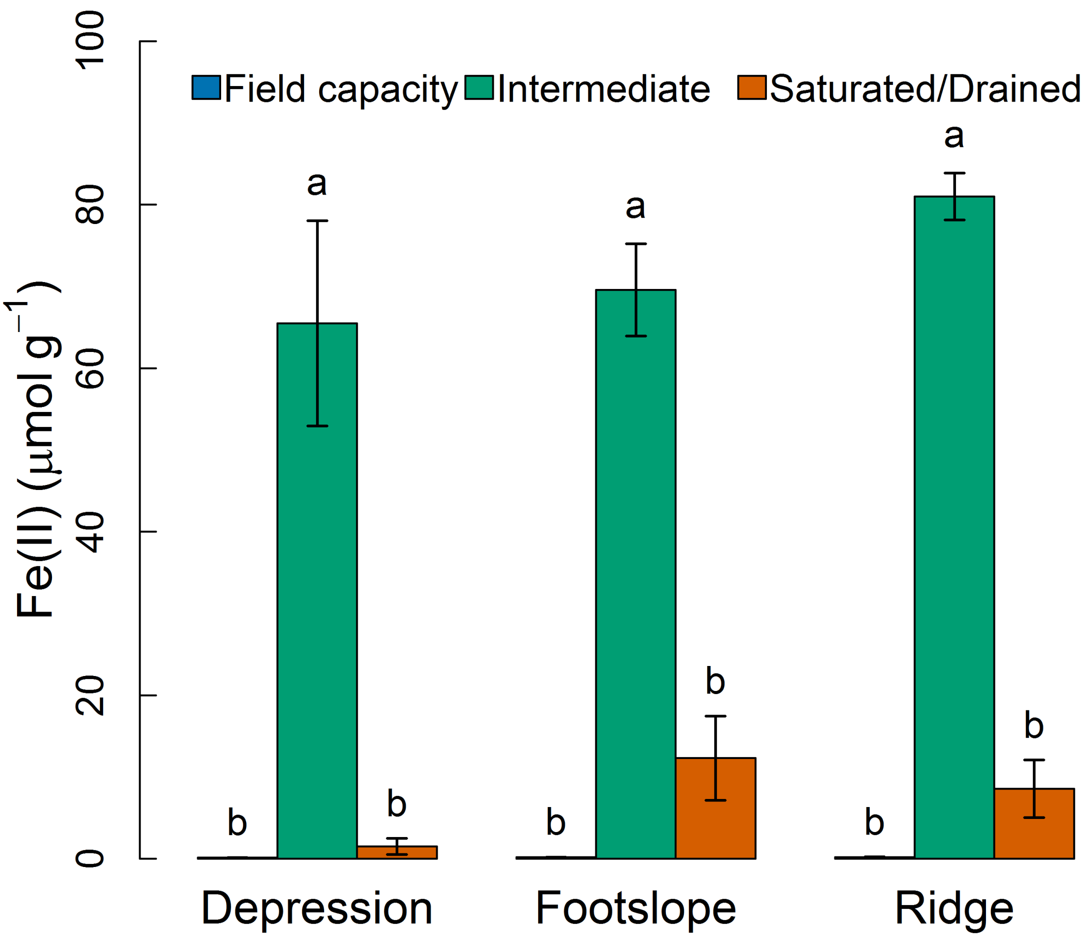

Supplementary Figure 9. Concentrations of ferrous iron in three Mollisols at the end of incubations. The error bars indicate s.e.m. (n = 4). The lowercase letters indicate significant differences between the moisture treatments within each soil type ($p < 0.05$).

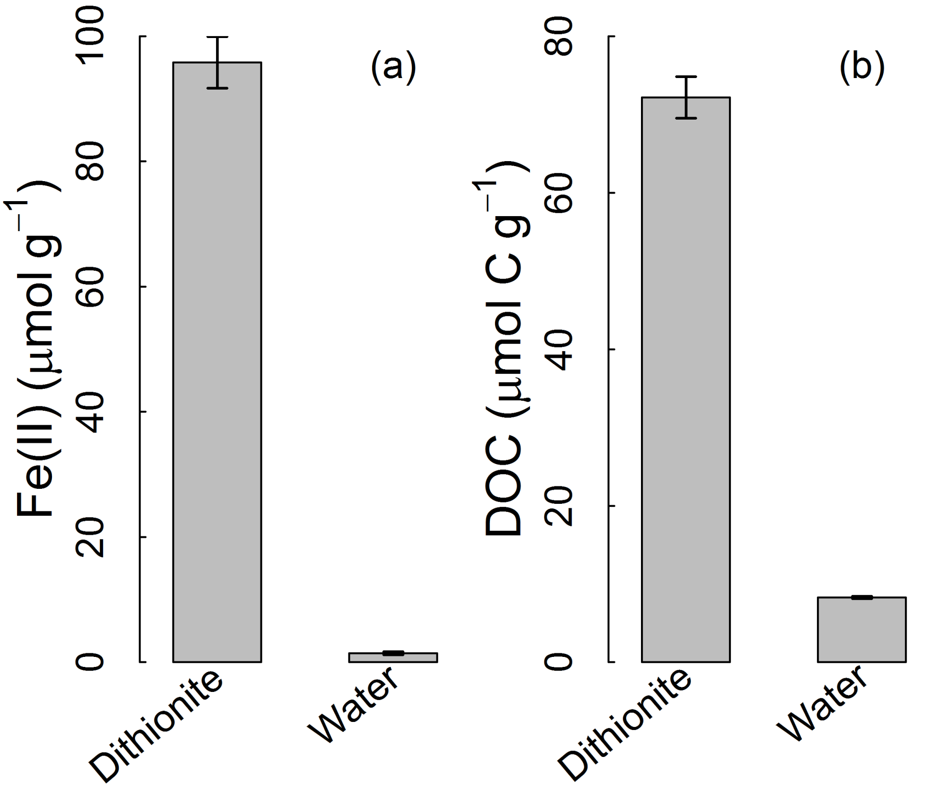

Supplementary Figure 10. Concentrations of ferrous iron and dissolved organic C in footslope soils extracted by inorganic dithionite and deionized water. (a) Ferrous iron (Fe(II)) concentration; (b) dissolved organic C (DOC) concentration. The error bars indicate s.e.m. (n = 5).

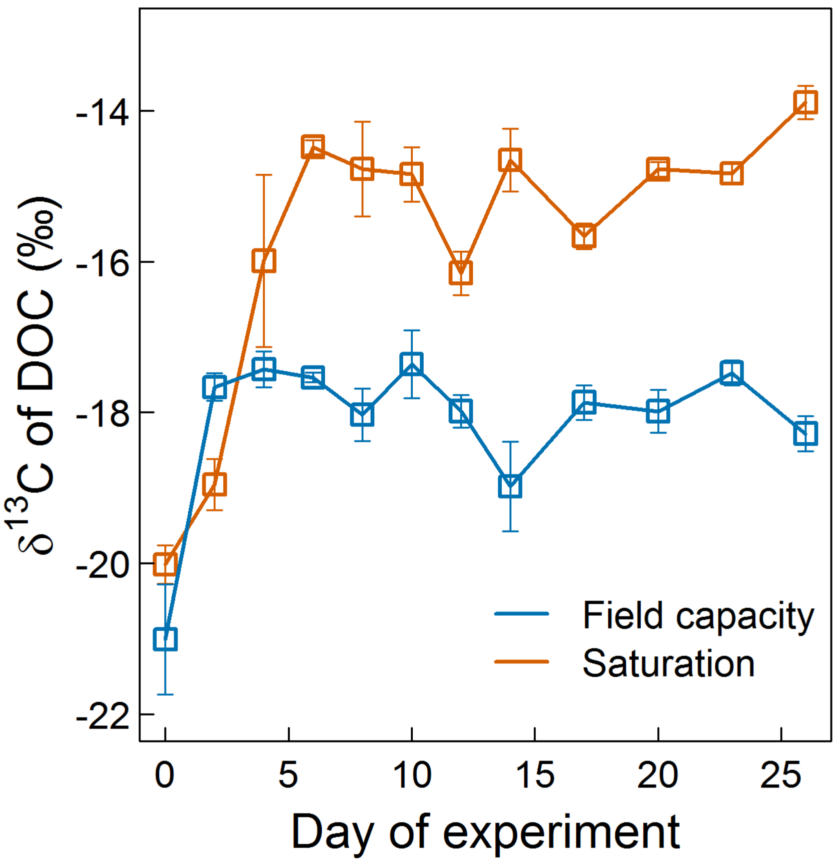

Supplementary Figure 11. Carbon isotope ratios of dissolved organic C in footslope soils incubated under field capacity and saturation. The error bars indicate s.e.m. (n = 4).

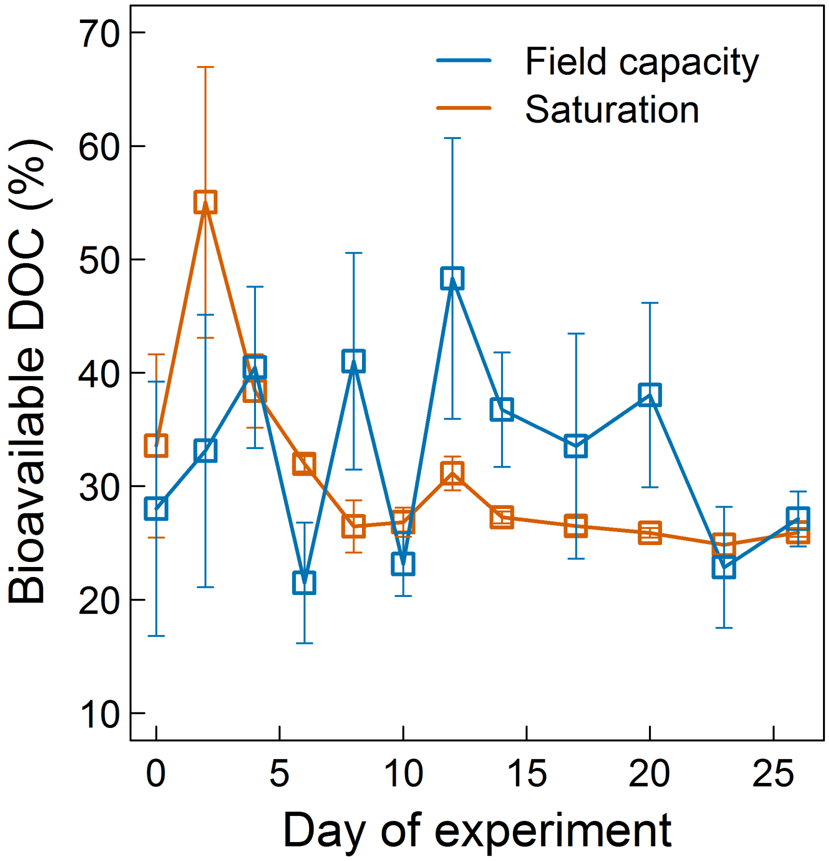

Supplementary Figure 12. Percentage of readily bioavailable DOC in footslope soils incubated under field capacity and saturation. Bioavailable DOC was measured during the additional incubation of the footslope soils, determined by incubating DOC samples with a microbial inoculum and measuring CO_2 production over five days, and normalizing by total DOC. The error bars indicate s.e.m. (n = 4).

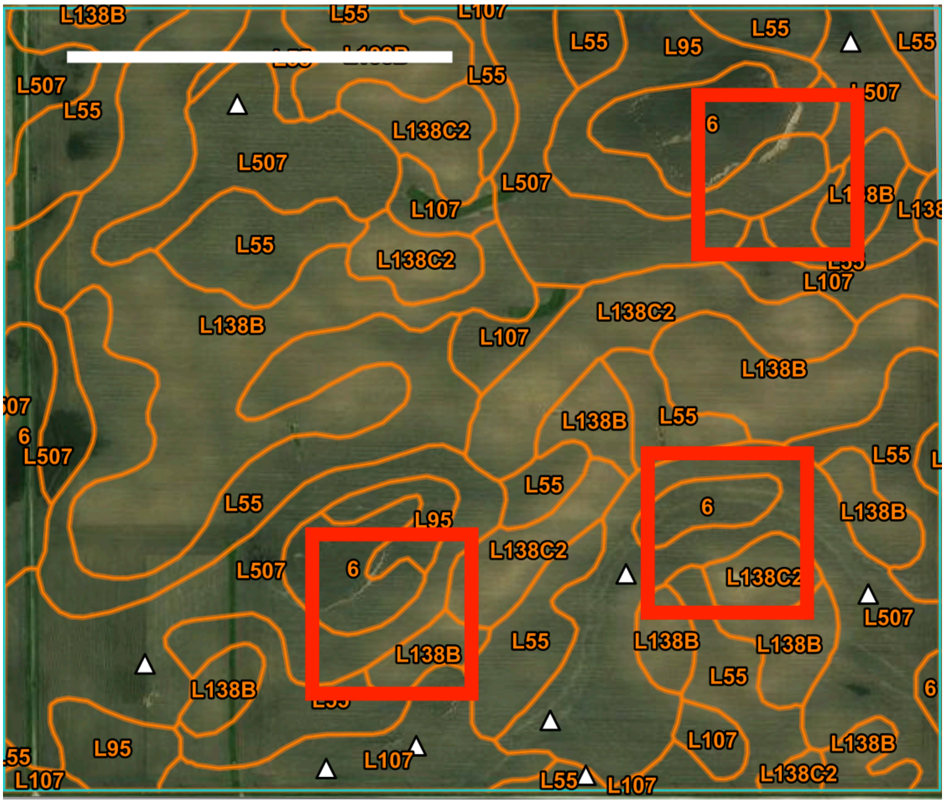

Supplementary Figure 13. Map of the field site. The locations of soil sampling (blocks) are denoted by red squares (200 m x 200 m), and USDA soil series are denoted by orange text and lines. The white line is a 500 m scale bar. The Okoboji (depression) soils are indicated by 6, Canisteo (footslope) soils are L507, and Clarion (ridge) soils are L138B. Each soil series was sampled from six locations within each block and composited with the same series from the other blocks to generate a spatially representative sample for each series from the field. The soil map was generated by the Natural Resources Conservation Service Web Soil Survey[1].

Supplementary Table 1 Soil physicochemical properties of the three Mollisols from 0 to 20 cm.

Soils	Soil texture			pH	Total C	$\delta^{13}C$ (‰)	Total N	C/N ratio	Fe_{ca}	Fe_{cd}
	Sand (%)	Silt (%)	Clay (%)		mg g^{-1}		mg g^{-1}		μmol g^{-1}	μmol g^{-1}
Ridge	43 ± 7	28 ± 4	29 ± 3	6.65 ± 0.58	18 ± 4	-21.85 ± 1.03	1.55 ± 0.34	12 ± 0	14.39 ± 3.24	119
Footslope	43 ± 3	30 ± 2	27 ± 1	6.98 ± 0.41	25 ± 2	-20.37 ± 0.15	2.03 ± 0.11	12 ± 0	15.55 ± 4.59	106
Depression	22 ± 7	40 ± 4	38 ± 3	6.98 ± 0.46	34 ± 6	-22.94 ± 0.52	2.73 ± 0.62	13 ± 1	17.07 ± 5.49	110

Values are means and s.e.m. (n = 3 except for Fe_{cd}, where n = 1). Fe_{ca}: citrate/ascorbate-extractable Fe; Fe_{cd}: citrate/dithionite-extractable Fe.

Supplementary References

1. USDA-NRCS. Web Soil Survey. https://websoilsurvey.sc.egov.usda.gov (2017).
